# Effect of enset (*Ensete ventricosum*) varieties and fermentation time on nutritional compositions, antinutritional factors, functional properties, and sensory acceptance of *Bulla*

**DOI:** 10.1002/fsn3.3632

**Published:** 2023-09-01

**Authors:** Hiwot Bekele, Kelbesa Urga, Habtamu Fekadu Gemede, Ashagrie Zewdu Woldegiorgis

**Affiliations:** ^1^ Centre for Food Science and Nutrition Addis Ababa University Addis Ababa Ethiopia; ^2^ Ethiopian Public Health Institute (EPHI) Addis Ababa Ethiopia; ^3^ Department of Food Technology and Process Engineering Wollega University Nekemte Ethiopia

**Keywords:** antinutrient, bulla, enset: varieties, fermentation, functional, mineral, sensory

## Abstract

The effect of enset varieties and fermentation time on the nutritional composition, antinutritional content, functional properties, and sensory acceptance of bulla was assessed. Bulla samples were prepared from newly improved enset varieties *yanbule*, *gewada*, *zereta*, and *messina* and were fermented for 0, 15, and 30 days. The result revealed that bulla prepared from *gewada* had relatively better values of fat (0.3 g/100 g), fiber (1.04 g/100 g), carbohydrate (97.7 g/100 g), energy (394.2 Kcal), and Fe (2.54 mg/100 g). *Yanbule* had relatively higher ash content (1.05 g/100 g) and considerably higher Ca (317.9 mg/100 g) than bulla prepared from the other varieties. Mg (56.8 mg/100 g) and Zn (2.3 mg/100 g) were relatively higher in bulla prepared from Messina. A very low level of tannin was detected only for *gewada*, but high contents of phytate up to 112.5 mg/100 g were obtained. With respect to the functional properties of bulla fermented for 30 days, no significant differences were observed among the varieties except for water absorption capacity. In terms of sensory quality, bulla porridge prepared from *yanbule* had comparatively higher overall acceptability score (7.6).

## Graphical abstract

Bulla is a processed starch or dough made from enset (*Enset ventricosum*), commonly known as the “false banana.” Enset contributes to the food security of approximately 15 million Ethiopians and helps in soil conservation. Therefore, enset‐derived foods are popular in the area. Bulla is a by‐product made from the creation of Enset, which is a paste made from the scraped enset pseudo‐stem and leaf sheaths, fermented for 10–15 days, and then steam‐baked into a flatbread. Bulla is obtained when the scrapings and pulp are squeezed, separating off the liquid. The starch that separates out from the liquid concentrates into a white powder: the bulla. This powder can be stored for years. It has a complex aroma, reminiscent of fresh cheese. To use bulla, it is first rehydrated with water to make dumplings, porridge, pancakes, or a beverage. Bulla is consumed mixed with traditional seasoned butter and spices, resulting in small grains that look like couscous. In Western Oromia, it is common to mix bulla powder with seasoned butter and fresh milk, cooked until it obtains a jelly‐like consistency.

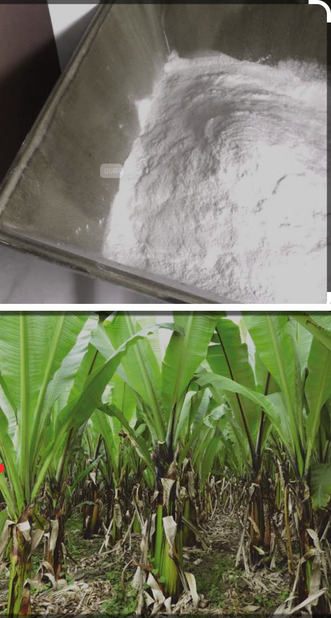

## 1 INTRODUCTION

Root and tuber crops are widely cultivated in southern Ethiopia and support a considerable portion of the country's population as source of food. Prominent among these are as follows: potato (*Solanum tuberosum L.)*, sweet potato (*Ipomoea batatas L*.), enset (*Ensete ventricosum* (Welw), Cheesman), godere (*Colocasia esculenta L*.), yams (*Dioscorea* spp.), Ethiopian dinch (*Coleus parviflorus*), koteharrie (*Diaspora bulbiferous*), and anchote (*Coccinia abyssinica*). Among these, enset is one of an endemic crop to Ethiopia (Aregahegn et al., 2013).

*Ensete ventricosum* (Welw.) Cheesman, commonly known as enset, is a monocarpic perennial herb that originated in Ethiopia (Olango et al., 2014). Bulla is a primary enset product that is obtained after processing (Forsido et al., 2013; Olango et al., 2014). Kocho and bulla foods are extremely popular at restaurants that serve the Ethiopian delicacy of Kitfo (raw minced beef mixed with butter and spice; Brand et al., 1997; Forsido et al., 2013).

Bulla is the liquid part obtained from the transformation process of enset into kocho and is considered as “the best part”. The internal part of the pseudostem is scraped with a piece of split bamboo in order to separate the starch from the fiber. The pulp is then pressed and collected as juice, which is then left to decant. This decantation generates the bulla, which is then fermented for many weeks to produce highly starchy food of the highest quality. It is only served on special occasions (religious, traditional feasts) or to honor guests (FCES, 2005). However, the nutritive value of starchy meals is determined primarily by their nutrient content as well as the presence of antinutritional factors and toxic compounds (Atlabachew, 2007; Treche, 1996). Recently, Areka Agricultural Research Center (AARC) released six enset varieties (*yanbule*, *gewada*, *endale*, *kelisa*, *zereta*, and *messina*) and registered by the Ministry of Agriculture and Rural Development, Animal and Plant Health Regulatory Department in 2009 and 2010 (Yeshitila & Yemataw, 2010).

There is growing interest in the role enset currently plays in regions of high consumption, but it remains an understudied crop in terms of exploring its challenges and potential for development (Macentee et al., 2013). Some research has been done on enset (Debebe, 2006; Forsido et al., 2013; Mohammed et al., 2013), containing the minerals (calcium, potassium, selenium, manganese, copper, zinc, chromium, cobalt, copper, nickel, phosphorus, iron, cadmium, and lead), crude protein, crude fiber, fat, and ash from Amicho, unfermented kocho, fermented kocho, unfermented bulla, fermented bulla, and on various parts of enset. In another study, Atlabachew (2007) determined the levels of major, minor, and trace elements in commercially available enset foods from the cities of Wolkite and Woliso. However, to the best of our knowledge, studies on the nutritional, antinutritional, and functional profile of the newly released enset varieties have not been conducted by the Areka Agricultural Research Center (AARC). Therefore, this study aimed to investigate the effect of enset varieties and fermentation varieties and fermentation time on nutritional composition, antinutritional factors, functional properties, and sensory acceptance of *Bulla*.

## 2 MATERIALS AND METHODS

### 2.1 Sample collection and preparation

A total of 16 kg of bulla was collected purposively from the four varieties of enset (*yanbule*, *zereta*, *messina*, and *gewada*). The samples were packed in polyethylene bags, kept in an ice box (to prevent moisture loss and fermentation), and transported to the Center for Food Science and Nutrition Laboratory of Addis Ababa University. The samples were divided into three lots/batches for various different fermentation times as soon as they arrived at the laboratory times. The first batch was used for analysis without fermentation, and the remaining two batches were fermented for 15 and 30 days before being analyzed for their nutritional and antinutritional content. The latter lots and batches were allowed to ferment in airtight plastic materials at room temperature under the same conditions until conditions for the required time. Prior to drying, the moisture content of each batch of lots was measured at the indicated fermentation time. Each of the three lots was dried in an oven at 60°C for 72 h before nutritional and antinutritional analysis. Before being stored in polyethylene bags until needed for analysis, each dried sample was ground into a fine powder using an electric grinder to pass through a 0.425 mm sieve mesh size (Fekadu et al., 2014).

### 2.2 Preparation of genfo and kofeme

Genfo (stiff porridge): The dried and milled bulla flour was used for the preparation of genfo, while mixing the flour in boiling water and was cooked until it retained the right consistency and flavor (Asrat, 2011).

Kofeme: To prepare kofeme (traditional food around Wolaita), which looks like injera firfir. The semidry bulla paste was spread on the hot girdle and mixed with a spoon until uniform pearls are formed. The kofeme was baked for about 15 min (Urga et al., 1993).

### 2.3 Proximate analysis

Moisture content of the sample was analyzed by drying air oven (DHG 9055A) method according to the official method 925.09 of the AOAC (2000). Protein content was determined by the Kjeldahl method according to the official method 979.09 of AOAC (2000). Crude fat test was carried out on Soxhlet extraction (SZC‐D fat determination meter YLC 2000) method utilizing diethyl ether according to official method 920.39 of the AOAC (2000). Crude fiber content was determined by using Fibertec (2010). The samples were analyzed by the steps of digestion, filtration, washing, drying, and combustion according to the official method 962.09 of the AOAC (2000). The ash content was measured according to the dry ashing procedure using the official method 923.03 of the AOAC (2000). The content of carbohydrates in the sample was determined by a difference method that is done by subtracting the sum of the percentages of moisture, crude protein, crude fat, crude fiber, and ash content from 100%.

### 2.4 Determination of mineral

Mineral (calcium, magnesium, zinc, and iron) analysis was determined by atomic absorption spectrophotometry (model AA‐6800 Shimadzu) after digestion with concentrated nitric acid (AOAC, 2000).

### 2.5 Determination of functional properties of bulla flours

#### 2.5.1 Determination of bulk density

The flour sample was filled to 5 mL into an already weighed measuring graduated cylinder (*W*_1_). For the packed bulk density determination, the flour sample was gently tapped to eliminate spaces between the flour and the level was noted to be the volume of the sample (*V*) and then weighed (*W*_2_). No tapping was made in the case of loosed bulk density and the level is also noted to be the volume of the sample and then weighed. The study was conducted in triplicate (Adebowale et al., 2005; Narayana & Narasinga, 1984).

#### 2.5.2 Determination of water absorption capacity (WAC)

WAC was determined by the method reported by Sosulski. Twenty‐five milliliters of distilled water was added to a sample of 3‐g flour (*W*_1_) in a weighed centrifuge tube (*W*_2_) and stirred six times for 1 min at 10‐min intervals. The mixture was centrifuged at 3000 rpm for 25 min and the clear supernatant was decanted and discarded. Pellets were dried at 50°C for 25 min and the adhered drops of water were removed and reweighed (*W*_3_). The amount of water retained in the sample was recorded as weight gain and was taken as water absorbed. Water absorption capacity was expressed as the weight of water bounded by 100‐g dried flour.

#### 2.5.3 Oil absorption capacity (OAC)

Ten milliliters of refined corn oil with a density of 0.92 g/cm^3^ was added to 1.0 g of the sample in a 25‐mL centrifuge tube. The suspension was stirred using a magnetic stirrer (Model MS‐12B, MS‐17B, and MS‐ 22B) for 5 min. The suspension obtained was centrifuged at 3555 rpm for 30 min and the supernatant was measured in a 10 ‐mL graduated cylinder. Oil absorbed will be calculated as the difference between the initial volume of oil added to the sample and the volume of the supernatant (Adebowale et al., 2005; Adeleke & Odedeji, 2010; Buchat 1977; Ocloo et al., 2010).

#### 2.5.4 Swelling power and solubility

Swelling power and solubility determination were carried out in the temperature range of 60–90°C (using the method of Leach et al., 1959). About 1 g of bulla flour sample was accurately weighed and transferred in a clear dried test tube and weighed (*w*_1_). About 10 mL of distilled water was added and mixed gently at low speed for 5 min. The slurry was heated in a thermostated water bath (YCW‐0125) at 80°C for 30 min and was stirred gently to prevent lumps forming in the flour. Then, the cooled test tube (20°C) was centrifuged at 2200 rpm for 15 min. The supernatant was decanted immediately after centrifuging into a preweighed evaporating can and at 100°C to a constant weight approximately for 4 h. The weight of the sediment was taken and recorded as *W*_2_ or the swollen.

### 2.6 Antinutritional analysis

Phytic acid was determined by using Latta and Eakin (1980) as modified by Vaintraub and Lapteva (1988). Condensed tannin was determined by the Vanillin assay of Burns (1971) as modified by Maxson and Rooney.

### 2.7 Sensory analysis

Bulla porridge and kofeme from each variety were presented to 10 trained panelists to evaluate the taste, color, appearance, flavor, and overall acceptability. The panelists were assigned to score their preference for the various attributes using 9‐point hedonic scale. With nine being like extremely and one dislike extremely (Urga & Umeta, 1993).

### 2.8 Experimental design and statistical analysis

The product was produced in triplicate and evaluated under completely randomized design (CRD). Data were analyzed by the analysis of variance (ANOVA) procedures using SPSS/20.0 software for windows. Least significant differences (LSD) were used for multiple mean comparison tests. Significance was accepted at (*p* < .05) level of probability.

## 3 RESULTS AND DISCUSSION

### 3.1 Proximate composition of bulla

The proximate composition of bulla among the four enset varieties (*yanbule*, *gewada*, *zereta*, and *messina*) is presented in Table 1, and also the effect of fermentation time (0, 15, and 30 days of fermentation) on the proximate composition of bulla is presented in Table 2.

**TABLE 1:** Proximate composition (g/100 g) of bulla prepared from different varieties of enset.

Varieties	Moisture	Protein	Fat	Ash	Fiber	Carbohydrate	Energy
Yanbule	52.5 ± 0.08^b^	0.19 ± 0.003b	0.29 ± 0.027a	1.05 ± 0.19a	1.02 ± 0.01a	97.45 ± 0.21a	393.19 ± 0.6a
Gewada	54.1 ± 0.45^a^	0.18 ± 0.005b	0.3 ± 0.027a	0.77 ± 0.06a	1.04 ± 0.005a	97.71 ± 0.03a	394.24 ± 0.37a
Zereta	49.57 ± 0.29^c^	0.36 ± 0.005a	0.23 ± 0.026a	0.91 ± 0.06a	1.04 ± 0.005a	97.45 ± 0.1a	393.35 ± 0.14a
Messina	48.55 ± 0.11^d^	0.36 ± 0.011a	0.29 ± 0.026a	0.77 ± 0.1a	1.04 ± 0.01a	97.54 ± 0.1a	394.21 ± 0.22a

*Note*: Value in each cell is mean ± SD duplicate analysis. Means in the same column followed by different superscript letters are significantly different (*p* < .05).

**TABLE 2:** The effect of fermentation on the proximate composition (g/100 g) of bulla.

Proximate composition	Varieties	Duration of fermentation		
		Raw	15 days	30 days
Moisture	Yanbule	52.5 ± 0.08^a^	51.1 ± 0.36^b^	49.87 ± 0.41^c^
	Gewada	54.1 ± 0.45^a^	50.97 ± 0.31^b^	50.27 ± 0.34^b^
	Zereta	49.57 ± 0.29^a^	48.38 ± 0.52^b^	47.88 ± 0.32^b^
	Messina	48.55 ± 0.11^a^	48.07 ± 0.17^a^	47.76 ± 0.91^a^
Crude protein	Yanbule	0.19 ± 0.003^c^	0.37 ± 0.005^b^	0.56 ± 0.01^a^
	Gewada	0.18 ± 0.005^c^	0.54 ± 0.01^b^	0.72 ± 0.016^a^
	Zereta	0.36 ± 0.005^c^	0.54 ± 0.013^b^	0.73 ± 0.005^a^
	Messina	0.36 ± 0.011^c^	0.58 ± 0.011^b^	0.91 ± 0.01^a^
Crude fiber	Yanbule	1.02 ± 0.01^a^	0.79 ± 0.029^b^	0.41 ± 0.006^c^
	Gewada	1.04 ± 0.005^a^	0.88 ± 0.03^b^	0.62 ± 0.012^c^
	Zereta	1.04 ± 0.005^a^	0.81 ± 0.012^b^	0.56 ± 0.056^c^
	Messina	1.04 ± 0.01^a^	0.85 ± 0.017^b^	0.42 ± 0.004^c^
Ash	Yanbule	1.05 ± 0.19^a^	1.24 ± 0.06^a^	1.27 ± 0.06^a^
	Gewada	0.77 ± 0.06^a^	0.78 ± 0.06^a^	0.88 ± 0.08^a^
	Zereta	0.91 ± 0.06^a^	0.92 ± 0.08^a^	1.14 ± 0.08^a^
	Messina	0.77 ± 0.1^a^	1.05 ± 0.25^a^	1.25 ± 0.04^a^
Crude fat	Yanbule	0.29 ± 0.027^a^	0.34 ± 0.026^a^	0.42 ± 0.052^a^
	Gewada	0.3 ± 0.027^b^	0.35 ± 0.027^ab^	0.45 ± 0.026^a^
	Zereta	0.23 ± 0.026^b^	0.4 ± 0.026^a^	0.47 ± 0.05^a^
	Messina	0.29 ± 0.026^a^	0.39 ± 0.027^a^	0.39 ± 0.027^a^
CHO	Yanbule	97.45 ± 0.21^a^	97.25 ± 0.002^a^	97.34 ± 0.01^a^
	Gewada	97.71 ± 0.03^a^	97.46 ± 0.11^ab^	97.34 ± 0.03^b^
	Zereta	97.45 ± 0.1^a^	97.33 ± 0.03^a^	97.11 ± 0.18^a^
	Messina	97.54 ± 0.1^a^	97.13 ± 0.28^a^	97.03 ± 0.004^a^
Energy kcal	Yanbule	393.19 ± 0.6^b^	393.57 ± 0.27^ab^	395.36 ± 0.48^a^
	Gewada	394.24 ± 0.37^b^	395.13 ± 0.23^ab^	396.23 ± 0.41^a^
	Zereta	393.35 ± 0.14^b^	395.05 ± 0.42^a^	395.58 ± 0.27^a^
	Messina	394.21 ± 0.22^a^	394.38 ± 0.94^a^	395.29 ± 0.3^a^

*Note*: Value in each cell is mean ± SD duplicate analysis. Means in the same row followed by different superscript letters are significantly different (*p* < .05).

### 3.2 Moisture content

The moisture content of bulla sample was significantly varied with the varieties of enset (Table 1). Bulla prepared from *gewada* (54.1%) had the highest moisture content and the least moisture content was recorded for bulla prepared from *messina* (48.6%). The moisture content of bulla prepared from *yanbule*, *gewada*, and *zereta* was significantly (*p* < .05) affected by fermentation time. The moisture content of bulla prepared from *yanbule* significantly varied from raw to fermented for 15 and 30 days. However, for bulla derived from *gewada* and *zereta*, moisture content was significantly reduced only after 15 and 30 days of fermentation. The significant variation in moisture content among the bulla samples greatly reflects the genetic difference among the varieties (Tobiaw & Bekele, 2011) and the variation in the degree of sedimentation during bulla preparation. The reason that the moisture content of fermented bulla was reduced could be because the microorganisms responsible for bulla fermentation might have utilized some moisture for their metabolic activities (Igbabul et al., 2014). The moisture contents of bulla reported in this study were in close agreement with the moisture content of commercially available bulla reported by Atlabachew (2007), which ranged from 44% to 55%. The moisture results also agreed with values reported for bulla (54.9%) in the Ethiopian Food Composition Table (EHNRI, 1997).

### 3.3 Protein content

The value of protein for the enset varieties is given in Table 1. The protein content of bulla significantly differed (*p* < .05) among the varieties. Bulla derived from *zereta* (0.36 g/100 g) and *messina* (0.36 g/100 g) had significantly higher protein contents than bulla from the rest of the varieties. The fermentation time brought significant increment in the protein content of bulla prepared from all varieties. The longer fermentation time (30) resulted in highest protein content for bulla. The protein content increased from 0.19 to 0.56 g/100 g for *yanbule*, 0.18–0.72 g/100 g for *gewada*, 0.36–0.73 g/100 g for *zereta*, and 0.36–0.90 g/100 g for *messina*. The protein contents obtained in this study were in agreement with the result of a study done by Atlabachew (2007) that ranged from 0.4 to 0.8 g/100 g but were higher than the values for bulla included in the Ethiopian Food composition table (0.2 g/100 g on dry matter basis; EHNRI, 1997).

The observed increase in protein content of fermented bulla samples could be attributed to the increase in microbial mass during fermentation, causing extensive hydrolysis of protein molecules to amino acid and other simple peptides. Secondly, the enzymatic hydrolysis of some protein inhibitors during fermentation, for instance, the degradation of antinutritional factors especially phytate may contribute to the increase in protein content due to the breakage of phytate–protein complexes. The increase may also be due to the structural proteins that are an integral part of the microbial cell (Igbabul et al., 2014). On the other hand, the variation in protein content of bulla could be a result of the differences in the genetic makeup of the enset varieties. According to Elyas et al. (2002) and Abdellaleem et al. (2008), the observed increase in protein content after fermentation can probably be due to the shift in dry matter content through depletion.

### 3.4 Total ash

The total ash, which is an indirect indicator of mineral content of foodstuff, varied between 0.77 and 1.05 g/100 g (Table 1). Although there were no considerable variations in ash contents of the bulla prepared from the four varieties, bulla from *yanbule* variety (1.05 g/100 g) had the highest ash content and the lowest ash value was recorded for bulla prepared from *gewada* (0.77 g/100 g) and *messina* (0.77 g/100 g). The relative variation in the ash content may largely be due to the differences in genetic composition and/or maturity level.

Fermentation on the other hand did not result in significant change in ash content of bulla prepared from all the four varieties of enset (Table 2). However, the ash content was high in bulla fermented for 30 days. The values of ash content in the current study were higher than the results reported by Atlabachew (2007), which was 0.2 g/100 g and were higher than the values presented in Ethiopian Food Composition Table, 0.1 g/100 g (EHNRI, 1997).

### 3.5 Crude fat

The values for crude fat content of raw bulla prepared from the four varieties were not significantly different (*p* > .05). Bulla prepared from *gewada* (0.30 g/100 g) had the highest fat content and was followed by bulla derived from *yanbule* (0.29 g/100 g*)* and *messina* (0.29 g/100 g; Table 1). Fermentation time did not significantly (*p* > .05) change the fat content for bulla prepared from *yanbule* and *messina* (Table 2). However, the fat content of bulla derived from *gewada* and *zereta* significantly varied with the fermentation time. The fat content of bulla prepared from *gewada* and *zereta* increased from 0.3 g/100 g to 0.45 g/100 g and 0.23 g/100 g to 0.7 g/100 g, respectively. The obtained result was comparable with the study by Atlabachew (2007) (0.2–0.4 mg/100 g). However, it was higher than the figure reported (0.1 g/100 g) in Ethiopian Food Composition Table (EHNRI, 1997). The increase in the fat content of bulla as the fermentation time extends could be attributed to the possibility that the fermenting microorganism could secrete microbial oil as a result of extensive breakdown of large fat molecules to simpler fatty acid units due to the high activity of the lipolytic enzymes (Igbabul et al., 2014). The decreased fat content of bulla may help to extend its shelf life because food products containing high fat are susceptible to both hydrolytic and oxidative or enzymatic rancidity and are responsible for both the general acceptability and storage stability of the product.

### 3.6 Crude fiber

The crude fiber content of the bulla derived from the four varieties seemed to be the same except for *yanbule* (1.02 g/100 g) which was slightly lower than others (1.04 g/100 g; Table 1). The fiber values reported in the present study were slightly higher than the value reported by Atlabachew (2007) (0.6–0.8 mg/100 g) and fiber content of 0.3 g/100 g reported in the Ethiopian Food Composition Table (EHNRI, 1997).

The fiber contents of the fermented bulla samples were substantially (*p* < .05) reduced for all varieties as the fermentation time became longer (Table 2). Fermentation decreased the fiber content of bulla by 40.4% (*gewada*) and 60.5% (*messina*). The least fiber content (0.41 g/100 g) was recorded for bulla prepared from *yanbule* and *messina* that was fermented for 30 days and fermented bulla derived from *gewada* had the highest fiber content (0.62 g/100 g). The observed decrease in the fiber content of bulla during fermentation could be attributed to the partial solubilization of cellulose and hemicellulose type of materials by microbial enzymes (Inyang & Zakari, 2008).

### 3.7 Utilizable carbohydrates

The multiple comparison tests showed no significant difference (*p* > .05) among bulla derived from the four varieties of enset in its carbohydrate content (Table 1). Bulla prepared from *gewada* had the highest carbohydrate content (97.71 g/100 g) and *zereta* and *yanbule* had the lowest carbohydrate content (97.45 g/100 g). The values were not different from the finding reported by Atlabachew (2007), which ranged from 93 to 98 g/100 g, but they were much higher than the value (44 g/100 g) reported in the Ethiopian food composition data table (EHNRI, 1997).

The current study indicated a decreasing pattern in the carbohydrate content during fermentation process in all the varieties. However, the reduction was not significant for all bulla products (Table 2). The reduction of the value as fermentation extends could be due to the fact that soluble starch and sugar are principal substances for fermenter microorganisms. Therefore, degradation and subsequent decrease in starch content are expected to occur. Fermentation will activate enzymes which act on polysaccharides. These enzymes degrade polysaccharides and the latter leads to the reduction of utilizable carbohydrates.

### 3.8 Gross energy

Table 1 shows that the total energy value ranged from 394.24 to 393.19 kcal/100 g. The highest and the lowest energy values were obtained from bulla prepared from *gewada* and *yanbule* varieties, respectively. The energy values reported in this research were in agreement with the findings of Atlabachew (2007) 378 to 442.6 kcal/100 g and much higher than the value of 180.5 kcal/100 g reported in the Ethiopian Food Composition Table (EHNRI, 1997).

### 3.9 The mineral composition of bulla

Calcium, magnesium, iron, and zinc contents of the four enset varieties (*yanbule*, *gewada*, *zereta*, and *messina*) are presented in Table 3.

**TABLE 3:** Mineral composition (mg/100 g) of bulla prepared from different raw varieties of enset.

Varieties	Ca	Mg	Fe	Zn
Yanbule	317.98 ± 2.54^a^	46.41 ± 1.13^a^	1.5 ± 0.026^b^	1.55 ± 0.39^ab^
Gewada	194.18 ± 6.42^c^	55.86 ± 0.14^a^	2.54 ± 0.117^a^	1.57 ± 0.25^ab^
Zereta	266.38 ± 2.79^b^	31.51 ± 4.23^b^	1.55 ± 0.154^b^	0.76 ± 0.05^b^
Messina	168.15 ± 7.94^d^	56.05 ± 2.26^a^	2.1 ± 0.184^a^	2.34 ± 0.05^a^

*Note*: Values are mean of duplicate ± SD. Means not sharing common superscript letters in a column are significantly different at *p* < .05.

#### 3.9.1 Calcium

The calcium contents of the unfermented bulla prepared from *yanbule* (317.98 mg/100 g) and *messina* (168.15 mg/100 g) had the highest and the least calcium content, respectively (Table 3). Bulla prepared from all varieties differed significantly (*p* < .05) from one another. This significant variation in the calcium content of bulla could be because of genetic differences among the enset varieties. Atlabachew (2007) reported the calcium content of bulla to be varied between 38.5 and 44.6 mg/100 g which was much lower than the value in this study. The amount of calcium (77 mg/100 g) which was presented in the Ethiopian food composition data table (EHNIR, 1997) was also much lower than the present value.

Fermentation time resulted in significant (*p* < .05) improvement of the calcium content of the bulla prepared from the four varieties. The highest calcium content for bulla fermented for 30 days was about 567.6 mg/100 g (*yanbule*) and the lowest calcium content for bulla fermented for 30 days was 239.95 mg/100 g (*messina*). The level of mineral depends on a number of factors including genetic property of the crop species, climatic conditions, soil characteristics, soil contamination, and the degree of maturity of the plant at the moment of harvesting. Moreover, the varieties were improved for their quality of product, yield of product, and shorting of the harvesting time (Yeshitila & Yemataw, 2010).

#### 3.9.2 Magnesium

The values for the level of magnesium content of raw bulla prepared from the four varieties were significantly different (*p* < .05). Bulla prepared from *messina* (56.05 g/100 g) had the highest magnesium content and was followed by bulla derived from *zereta* (31.51 g/100 g; Table 3). Fermentation time did significantly (*p* > .05) change the magnesium content for bulla prepared from *yanbule* and *zereta*. However, the magnesium content of bulla derived from *gewada* and *messina* was not significantly varied with the fermentation time. The magnesium content of bulla prepared from *yanbule* and *zereta* increased from 46.41 g/100 g to 68.01 g/100 g and 31.51 g/100 g to 57.54 g/100 g, respectively. The obtained result was higher than the study done by Atlabachew (2007) (58.4–89.5 μg/g).

The difference in the magnesium content of the current study and the previous one may be due to genetic makeup variation, although the plants have grown in the same environmental condition. Moreover, these four varieties of enset covered by the current study and the additional varieties (endale and kelisa) are the improved varieties for quality and kocho yield (Yeshitila & Yemataw, 2010). It directly influences the mineral content of bulla.

#### 3.9.3 Iron

Iron was another mineral analyzed in this study. The level of iron contents of the unfermented bulla prepared from *gewada* (2.54 mg/100 g) and *yanbule* (1.5 mg/100 g) had the highest and the least iron content, respectively (Table 3). Bulla prepared from *yanbule* and *zereta* varieties differed significantly (*p* < .05) from bulla prepared from *gewada* and *messina* varieties. This significant variation in the iron content of bulla could be because of genetic differences among the enset varieties and degree of maturity of the harvested enset. Atlabachew (2007) reported the iron content of bulla to be varied between 3.65 and 5.98 mg/100 g on dry matter base, which was much higher than the value in this study. The amount of iron (2.6 mg/100 g) which was presented in the Ethiopian Food Composition Table (EHNIR, 1997) was in line with the present value.

Fermentation time resulted in significant (*p* < .05) improvement of the iron content of the bulla prepared from the *yanbule* variety, whereas bulla prepared from *gewada*, *zereta*, and *messina* were not significantly (*p* > .05) affected by the duration of fermentation. The highest iron content for bulla fermented for 30 days was about 3.01 mg/100 g (*yanbule*) and the lowest iron content for bulla fermented for 30 days was 1.85 mg/100 g (*zereta*).

#### 3.9.4 Zinc

The value of zinc content for the studied enset varieties is given in Table 3. The level of zinc content of bulla significantly differed (*p* < .05) between *zereta* and *gewada* varieties. Bulla derived from *messina* (2.34 mg/100 g) and *zereta* (0.76 mg/100 g) had significantly higher and least zinc contents, respectively. The fermentation time brought significant increment in the zinc content of bulla prepared from all varieties. There was a significant difference (*p* < .05) in the zinc content of fermented bulla samples among the varieties. The longer fermentation time (30) resulted in highest level of zinc content for bulla. The zinc content increased from 1.55 to 3.81 mg/100 g for *yanbule*, 1.57–3.19 mg/100 g for *gewada*, 0.76–1.41 mg/100 g for *zereta*, and 2.34–5.43 mg/100 g for *messina*. The zinc contents obtained in this study were comparable with the result of study done by Atlabachew (2007) that ranged from 2.2 mg/100 g to 4.43 mg/100 g. The possible explanation for the increment of zinc content of bulla obtained from *messina* variety might be contamination from the soil, water used for squeezing, or contamination of samples from enset leaves.

### 3.10 The antinutritional (phytate and tannin) factor content of bulla

The phytate and tannin contents of the four enset varieties (*yanbule*, *gewada*, *zereta*, and *messina*).

#### 3.10.1 Phytate

The values for the phytate content of raw bulla prepared from *yanbule* and *messina* varieties were significantly different (*p* < .05) from *gewada* and *zereta*. Bulla prepared from *yanbule* (112.56 mg/100 g) had the highest phytate content and was followed by bulla derived from *messina* (104.35 mg/100 g; Table 4). Fermentation time significantly (*p* < 0.05) changed the phytate content for bulla prepared from all the varieties. The phytate content of bulla prepared from *yanbule*, *gewada*, *zereta*, and *messina* decreased from 112.56 mg/100 g to 11.22 mg/100 g, 86.16 mg/100 g–10.66 mg/100 g, 84.25 mg/100 g–14.7 mg/100 g, and 104.35 mg/100 g–9.27 mg/100 g, respectively. Fermentation reduced the phytate by 88% in the current study. This is in agreement with the previous studies on cassava varieties (Abera et al., 2013), which resulted as fermentation reduces phytate content by 88.4%. Reduction in the phytate content of cereals with processing treatment has been frequently reported by Ibrahim et al. (2002). This has been attributed to an increase of phytase activity on the phytate that makes the phytate soluble and releases bound protein and minerals. The reduction may be attributed to the activity of the endogenous phytase enzyme from the raw ingredient and inherent microorganisms which are capable of hydrolyzing phytic acid in fermented food preparations into inositol and orthophosphate (Igbabul et al., 2014).

**TABLE 4:** The phytate and tannin contents of bulla prepared from different raw varieties of enset.

Antinutrients	Varieties	Duration of fermentation		
		Raw	15 days	30 days
Phytate	Yanbule	112.56 ± 1.95^a^	14.43 ± 0.25^b^	11.22 ± 0.1^b^
	Gewada	86.16 ± 0.63^b^	19.87 ± 0.12^a^	10.66 ± 0.18^b^
	Zereta	84.25 ± 0.23^b^	19.46 ± 0.65^a^	14.7 ± 0.16^a^
	Messina	104.35 ± 3.31^a^	14.89 ± 0.44^b^	9.72 ± 0.29^c^
Tannin	Yanbule	BDL	BDL	BDL
	Gewada	0.01 ± 0.003	BDL	BDL
	Zereta	BDL	BDL	BDL
	Messina	BDL	BDL	BDL

*Note*: Mean value ± SD *n* = 3. Means within the same column followed by same letters are not statistically significant (*p* < .05).

*Abbreviation*: BLD, below the detected level.

#### 3.10.2 Condensed tannins

The tannin content of *yanbule*, *zereta*, and *messina* in all fermentation time was below the detected level. However, raw bulla prepared from *gewada* has 0.01 mg/100 g of tannin (Table 4). The decrease in the tannin content of bulla is acceptable because the higher content of tannins is known to inhibit the activity of the digestive enzymes and thus, interfere with the digestion and absorption of dietary protein, carbohydrates, minerals, and other nutrients. They may also cause damage to the mucosa of the digestive tract and hence, they are undesirable for human consumption from a nutritional point of view (Vijayakumari et al., 2007).

### 3.11 Functional properties of bulla flour

The functional properties of bulla flour from the four varieties of enset are shown in Table 5. The analysis was done on *Yanbule*, *Gewada*, *Zereta*, *and Messina* varieties of bulla which are fermented for 30 days. Because bulla fermented for 30 days had better nutritional value when it is compared with unfermented and 15 days fermented bulla. The functional properties covered by the current study are bulk density, water absorption capacity, oil absorption capacity, and swelling power.

**TABLE 5:** Effect of variety on functional properties of bulla fermented for 30 days.

Varieties	OAC (mL/g)	WAC (%)	BD (g/mL)	SP (%)
Yanbule	0.85 ± 0.15^a^	118.75 ± 0.42^c^	1.01 ± 0.01^a^	1.65 ± 0.01^a^
Gewada	0.9 ± 0.1^a^	114.17 ± 1.67^c^	1.01 ± 0.01^a^	1.62 ± 0.004^a^
Zereta	0.6 ± 0.1^a^	130 ± 4.17^b^	0.99 ± 0.01^a^	1.62 ± 0.05^a^
Messina	0.7 ± 0.1^a^	141.67 ± 0.83^a^	1.01 ± 0.01^a^	1.6 ± 0.01^a^

*Note*: Results are mean value of triplicate ± SD. Means with different superscript letters within the column are significantly different (*p* < .05).

*Abbreviations*: BD, bulk density; OAC, oil absorption capacity; SP, swelling power; WAC, water absorption capacity.

#### 3.11.1 Bulk density

The bulk density of bulla flour is presented in Table 5. The results show that the bulk density of bulla flour for *yanbule*, *gewada*, *zereta*, and *messina* was 1.01, 1.01, 0.99, and 1.01 g/mL, respectively. There was no significant difference among the four varieties (*p* > .05) in their bulk density. The value of bulk density determined in this study is close to the bulk density of cassava flour reported by Abera et al. (2013) that ranged from 0.65 to 1.70 g/mL. As compared to the bulk density of potato flour reported by Chandra and Samsher (2013), which is 0.72 g/mL, the value obtained from the current study is higher and can be chosen as good quality of flour. Bulk density of flour is related to the textural characteristics and ease of rehydration. Bulk density is an indication of the porosity of a product, which influences package design and could be used in determining the type of packaging material required for the product (Solsuki, 1962).

#### 3.11.2 Water absorption capacity

The water absorption capacity of the bulla flour among the varieties is presented in Table 5. The water absorption capacity of bulla flour for *yanbule*, *gewada*, *zereta*, and *messina* was 118.78, 114.17, 130, and 141.67%, respectively. The water absorption of each variety has significant difference (*p* < .05) except for *yanbule* and g*ewada* no significant difference between them. The result obtained by this study was much lower than the water absorption capacity of potato flour (752%) as reported by Chandra and Samsher (2013). Water absorption capacity describes flour–water association ability under limited water supply. It has been observed to be dependent on the starch and protein concentration in the material coupled with the size of the particle. Generally, the water absorption characteristics of root flour is very important depending on the ultimate product to which the flour is intended to be converted, such as snack foods and bakery products.

#### 3.11.3 Oil absorption capacity

The oil absorption capacity of *yanbule*, *gewada*, *zereta*, and *messina* was 0.85, 0.9, 0.6, and 0.7 g/g, respectively (Table 5). It ranges from 0.6 to 0.9 mL/g, where the highest value is recorded for *gewada* and the lowest for *zereta*. In contrast, the oil absorption capacity of cassava flour is 0.65–1.9 mL/g (Abebe et al., 2007).

#### 3.11.4 Swelling power

The swelling power of bulla flour, which is fermented for 30 days among the varieties was 1.65, 1.62, 1.62, and 1.6% for *yanbule*, *gewada*, *zereta*, and *messina*, respectively (Table 5). The results obtained from the current study are lower than the value of 42.9% reported for potato (Chandra & Samsher, 2013).

As swelling refers to absorption of water, it may be inhibited by the lipid content of the flour. Swelling power of the flour depends on the capacity of the flour to hold water through hydrogen bonding. After fermentation, the hydrogen bond of the starch molecules in the flour will be broken and replaced by hydrogen bonds with water (Abera et al., 2013).

### 3.12 Sensory analysis

The data from this experiment indicate that there was relationship between the bulla quality parameters and sensory evaluation results. The sensory analysis was done on y*anbule*, g*ewada*, *zereta*, and *messina* varieties of bulla which are fermented for 30 days. Bulla fermented for 30 days had better nutritional value when it is compared with unfermented and 15‐day fermented bulla.

#### 3.12.1 Sensory analysis for porridge

The prepared bulla porridge had good taste, color, texture, aroma, and overall acceptability. The highest mean score for porridge color (8.6), texture (8.9), taste (8.4), aroma (7.2), and overall acceptability (7.6) was for *yanbule* except the aroma of *messina*. The lowest mean for color (7.1), texture (7.2), taste (6.6), aroma (5.7), and overall acceptability (6.9) was obtained for *zereta* except texture to *messina*. As the fermentation time of bulla increased, its color acceptance also increased (Table 6). The fermenting microorganisms degrade some of the complex carbohydrates, and pigments from the cellular structure of pulp, by producing active enzymes. With regard to the taste, as the fermentation time of bulla increased, its flavor level also increased. Bulla porridge prepared from *yanbule* variety showed good sensory profile and overall acceptability and the reverse is true for *zereta* variety.

**TABLE 6:** Sensory quality of fermented bulla for 30 days among varieties.

Varieties	Sensory attributes’				
	Taste	Color	Aroma	Texture	Overall acceptability
Porridge					
Yanbule	8.4 ± 0.52^a^	8.6 ± 0.52^a^	7.1 ± 2.13^a^	8.9 ± 0.32^a^	7.6 ± 2.27^a^
Gewada	6.7 ± 2.12^a^	8 ± 1.89^a^	6.1 ± 2.42^a^	7.9 ± 1.85^a^	7.4 ± 1.26^a^
Zereta	6.6 ± 2.32^a^	7.1 ± 1.91^a^	5.7 ± 2.67^a^	7.7 ± 2.26^a^	6.9 ± 2.32^a^
Messina	8 ± 1.33a	8.5 ± 0.97^a^	7.2 ± 2.15^a^	7.2 ± 1.99^a^	7.5 ± 2.42^a^
Kofeme					
Yanbule	9.0 ± 0.00^a^	8.7 ± 0.95^a^	8.2 ± 1.23^a^	8.4 ± 1.26^a^	8.6 ± 0.52^a^
Gewada	8.9 ± 0.32^a^	8.8 ± 0.42^a^	8.1 ± 0.74^a^	8.2 ± 1.03^a^	8.6 ± 0.52^a^
Zereta	8.4 ± 0.97^a^	8.7 ± 0.67^a^	8.1 ± 1.52^a^	8.6 ± 1.26^a^	8.5 ± 0.97^a^
Messina	8.7 ± 0.48^a^	8.5 ± 0.97^a^	8.3 ± 1.25^a^	8.4 ± 1.26^a^	8.6 ± 0.97^a^

#### 3.12.2 Sensory analysis for kofeme

Kofeme prepared from the fermented bulla varieties had good taste, color, flavor, texture, and overall acceptability. As the result indicated in Table 6, the highest value was recorded 9.0 for taste, 8.8 for color, 8.3 for aroma, and 8.6 for texture for bulla prepared from *yanbule*, *gewada*, *messina*, and *zereta*, respectively. The overall acceptability of kofeme prepared from *yanbule*, *gewada*, and *messina* had the highest mean score (8.6). When the overall acceptability of kofeme is compared to the overall acceptability of porridge, kofeme had better acceptance.

## 4 CONCLUSION

The high concentration of phytate in bulla can have deleterious effects on the absorption of nutrients and micronutrients and also may interfere with the function of certain organs. The presence of antinutritional factors in bulla products may limit its utilization for human consumption. Combining all the results of this study, the most commonly used traditional method of processing had resulted in a tremendous reduction of the moisture, fiber, pH, phytate, and tannin contents of bulla. Moreover, it also enhanced the protein, fat, ash, carbohydrate, total energy, and mineral of the raw sample. In conclusion, extending the fermentation time and different enset varieties had resulted in differences in chemical composition and sensory attributes of bulla. Bulla fermented for 30 days showed better nutritional quality and lower antinutritional factors. Bulla prepared from *yanbule* and *messina* varieties was better in physic‐chemical properties, chemical composition, and sensory acceptability. However, these two varieties had a higher phytate content, which may affect the utilization of nutrients by the body. Generally, the selection of appropriate varieties and use of recommended fermentation time were found to be good practice for the processing of traditional enset products such as bulla which is a traditional fermented food in Ethiopia.

## AUTHOR CONTRIBUTIONS

**Habtamu Fekadu Gemede:** Data curation (lead); formal analysis (lead); investigation (lead); software (lead); supervision (lead); writing – original draft (lead); writing – review and editing (lead). **Hiwot Bekele:** Conceptualization (lead); data curation (lead); funding acquisition (lead); supervision (lead); writing – original draft (supporting). **Kelbesa Urga:** Conceptualization (lead); methodology (lead); writing – review and editing (lead). **Ashagrie Zewdu Woldegiorgis:** Formal analysis (lead); investigation (supporting); resources (lead); validation (lead); writing – original draft (lead); writing – review and editing (supporting).

## CONFLICT OF INTEREST STATEMENT

The authors declare that that have no conflict of interest.

## Data Availability

All the required data will be made available upon request.

## References

[fsn33632-bib-0001] Abebe, Y. , Bogale, A. , Hambidge, K. M. , Stoecker, B. J. , Bailey, K. , & Gibson, R. S. (2007). Phytate, zinc, iron and calcium content of selected raw and prepared food consumed in rural Sidamo, Southern Ethiopia and implication for bioavailability. Journal of Food and Composition Analysis, 20, 161–168.

[fsn33632-bib-0102] Abera, T. T. , Admassu, S. E. , Desse, G. H. , & Bekele, T. G. (2013). Effect of processing on physicochemical composition and anti‐nutritional factors of cassava (Manihot Esculentacrantz) grown in Ethiopia. International Journal of Science Innovations and Discoveries, 3(2), 212–222.

[fsn33632-bib-0101] Abdellaleem, W. E. , El jirnar, A. A. , Mustafa, A. I. , & Babiker, E. E. (2008). Effect of fermentation on cereals pre‐treatment and cooking on anti‐nutritional factors and protein digestibility of cereals. Pakistan Journal of Nutrition, 7, 314–320.

[fsn33632-bib-0002] Adebowale, Y. A. , Adeyemi, I. A. , & Oshodi, A. A. (2005). Functional and physicochemical properties of flours of six Mucuna species. Journal of Biotechnology, 4(12), 1461–1468.

[fsn33632-bib-0003] Adeleke, R. O. , & Odedeji, J. O. (2010). Functional properties of wheat and sweet potato flour blends. Pakistan Journal of Nutrition, 9(6), 535–538.

[fsn33632-bib-0006] AOAC . (2000). Official method of analysis (vol. II 17th edition) of AOAC international Washington, DC, USA. Association of official analytic chemists.

[fsn33632-bib-0007] Aregahegn, A. , Chandravanshi, B. S. , & Atlabachew, M. (2013). Levels of major, minor and toxic metals in tubers and flour of Dioscorea abyssinica grown in Ethiopia. African Journal of Food, Agriculture, Nutrition and Development, 13, 7870–7887.

[fsn33632-bib-0010] Asrat, W. (2011). Development of suitable process for some Ethiopian traditional foods using quality protein maize, Ethiopian health and nutrition research institute (EHNRI), Proceeding of the Third National Maize Workshop of Ethiopia, pp 260–267.

[fsn33632-bib-0011] Atlabachew, M. (2007). Studies on commercially available enset (Ensete ventricosum (Welw.), Cheesman) food products (kocho and bulla) for major, minor and trace elements. Addis Abeba University.

[fsn33632-bib-0013] Brand, S. A. , Spring, A. , Hiebsch, C. , Mccabe, J. T. , Tabogie, E. , Diro, M. , Wolde‐Michael, G. , Yntiso, G. , Shigeta, M. , & Tabogie, E. (1997). The tree against hunger: Enset based agricultural system in Ethiopia (pp. 1–150). American Association for the Advancement of Science with Hawassa agricultural research Centre.

[fsn33632-bib-0014] Buchat, L. R. (1977). Functional and electrophoretic characteristics of succinylated peanut flour proteins. Journal of Agricultural and Food Chemistry, 25, 258–261.

[fsn33632-bib-0015] Chandra, K. , & Samsher, D. (2013). Assessment of functional properties of different flours. African Journal of Agricultural Research, 8(38), 4849–4852.

[fsn33632-bib-0017] Debebe, A. (2006). Studies of enset (Enset ventricosum) for major, minor and trace elements. Addis Ababa University School of Graduate Studies.

[fsn33632-bib-0104] EHNRI . (1997). Food composition table for use in Ethiopia (part III). Ethiopian Health and Nutrition Research Institute.

[fsn33632-bib-0105] Elyas, H. A. S. , El jinar, H. A. , & Yousef, E. N. (2002). Effect of natural fermentation on nutrition value and in vitro protein digestibility of cereals. Journal of Food Chemistry, 78, 75–79.

[fsn33632-bib-0023] Fekadu, H. , Beyene, F. , & Haki, G. D. (2014). Evaluation of bioavailability and sensory preference of processed Anchote (*Coccinia Abyssinica*) tubers in eastern Wollega. Ethiopia Journal of Food and Nutrition Sciences, 2(1), 1–12.

[fsn33632-bib-0024] Forsido, S. F. , Rupasinghe, V. , & Astatkie, T. (2013). Antioxidant capacity, total phenolic and nutritional content in selected Ethiopian staple food ingredients. International Journal of Food Sciences and Nutrition, 64(8), 915–920. 23777527 10.3109/09637486.2013.806448

[fsn33632-bib-0025] French Centre of Ethiopian Studies FCES Report . (2005). Analysis diagnosis of an agricultural region of Southern Ethiopia (Kembata, Homa kebele). FCES.

[fsn33632-bib-0106] Ibrahim, S. S. , Habiba, R. A. , Shatta, A. A. , & Embaby, H. E. (2002). Effect of soaking germination, cooking and fermentation on anti‐nutrition factors in cereals cowpeas. Nahrung, 46, 92–95. 12017999 10.1002/1521-3803(20020301)46:2<92::AID-FOOD92>3.0.CO;2-P

[fsn33632-bib-0107] Igbabul, B. D. , Amove, J. , & Twadue, I. (2014). Effect of fermentation on the proximate composition, antinutritional factors and functional properties of cocoyam (*Colacasia esculanta*) flour. African Journal of Food Science and Technology, 5(3), 67–74.

[fsn33632-bib-0108] Inyang, C. U. , & Zakari, V. M. (2008). Effect of fermentation on cereals on proximate chemical and sensory properties. Pakistan Journal of Nutrition, 7, 9–12.

[fsn33632-bib-0033] Latta, M. , & Eakin, M. (1980). Simple and rapid colorimetric method for phytate determination. Journal of Agricultural and Food Chemistry, 28, 1315–1317.

[fsn33632-bib-0034] Leach, H. W. , McCowen, L. D. , & Schoch, T. J. (1959). Structure of the starch granules, in: Swelling and solubility patterns of various starches. Cereal Chemistry, 36, 534–544.

[fsn33632-bib-0035] Macentee, K. , Thompson, J. , Fikreyesus, S. , & Jihad, K. (2013). Gender and Enset in Jimma zone, Ethiopia. Ethiopian Journal of Applied Science and Technology, 8(Special Issue No.1), 103–109.

[fsn33632-bib-0037] Mohammed, B. , Gabel, M. , & Karlsson, L. M. (2013). Nutritive values of the drought tolerant food and fodder crop enset. African Journal of Agricultural Research, 8(20), 2326–2333.

[fsn33632-bib-0039] Narayana, K. , & Narasinga, R. (1984). Effect of partial hydrolysis on winged bean flours. Journal of Food Science, 49, 944–947.

[fsn33632-bib-0041] Ocloo, F. C. K. , Bansa, D. , Boatin, R. , Adom, T. , & Agbemavor, W. S. (2010). Physicochemical, functional and pasting characteristics of flour produced from jackfruits (*Artocarpus Heterophyllus*) seeds. Agriculture and Biology Journal of North America, 1(5), 903–908.

[fsn33632-bib-0042] Olango, T. M. , Tesfaye, B. , Catellani, M. , & Enrico, P. M. (2014). Indigenous knowledge, use and on farm management of enset (*Ensete ventricosum* (Welw.) Cheesman) diversity in Wolaita, Southern Ethiopia. Journal of Ethnobiology and Ethnomedicine, 10, 41. 24885715 10.1186/1746-4269-10-41 PMC4025559

[fsn33632-bib-0109] Tobiaw, D. C. , & Bekele, E. (2011). Analysis of genetic diversity among cultivated enset (*Ensete ventricosum*) populations from Essera and Kefficho, south western part of Ethiopia. African Journal of Biotechnology, 10, 15697–15709.

[fsn33632-bib-0048] Treche, S. (1996). Tropical root and tuber crops as human staple wood, Confirenee presenteeau I Congresso Latino americano de Raizes Tropicals, 7–10 Octobre, Sao Pedro ‐ SP Bresil, France.

[fsn33632-bib-0050] Urga, K. , Nigatu, A. , & Umeta, M. (1993). Traditional enset based foods: Survey of processing techniques in Sidama. Ethiopian Nutrition Institute.

[fsn33632-bib-0110] Urga, K. , & Umeta, M. (1993). Traditional enset based foods: Survey of processing techniques in Sidama. Ethiopian Nutrition Institute.

[fsn33632-bib-0051] Vaintraub, I. A. , & Lapteva, N. A. (1988). Colorimetric determination of phytate in unpurified extracts of seeds and the products of their processing. Analytical Biochemistry, 175, 227–230. 3245569 10.1016/0003-2697(88)90382-x

[fsn33632-bib-0103] Vijayakumari, K. , Pugalenthi, M. , & Vadivel, V. (2007). Effect of soaking and hydrothermal processing methods on the levels of ant nutrients and in vitro protein digestibility of Bauhini purpureal seed. Journal of Food Chemistry, 103, 968–975.

[fsn33632-bib-0052] Yeshitila, M. , & Yemataw, Z. (2010). Past research achievement and existing gaps on enset *(Ensete ventricosum* (Welw.) Cheesman) breading, Areka agricultural research Centre (AARC), Ethiopia.

